# Is bipolar mixed depression associated with a good response to psychotropic augmentation?

**DOI:** 10.1192/j.eurpsy.2024.100

**Published:** 2024-08-27

**Authors:** Z. Rihmer

**Affiliations:** Psychiatry and Psychotherapy, Semmelweis University, Budapest, Hungary

## Abstract

**Abstract:**

Is bipolar mixed depression associated with a good response to psychotropic augmentation? Zoltán Rihmer Department of Psychiatry and Psychotherapy, Semmelweis University, Budapest and at the National Institute of Mental Helath, Neurology and Neurosurgery, Budapest, Hungary.

**Introduction:**

Suboptimal response to antidepressant pharmacotherapy (nonresponse or partial response but no remisson) is the most challenging issue in the treatment of depressive disorders. Open and controlled clinical studies show that augmentation of the given antidepressant with lithium, atypical antipsychotics, antiepileptics and thyroid hormones are effective in 30-40% in such cases.

**Objectives:**

To explore the possiblity wether bipolar mixed depression is the ideal subject of good response to psychotroic augmentation.

**Method:**

Literature review.

**Results:**

Studies consistently indicate that in contrast to unipolar MDE (=MDD) the rate of antidepressant-resistant depression is higher not only in bipolar I and II depression but also in MDE with subthreshold bipolarity (bipolar mixed depression). However, lithium, atypical antipsychotic and antiepileptic (but not thyroid) augmentation works much better in bipolar depression and in unipolar MDE with subthreshold bipolarity (mixed depression) tjan in unipolar MDE without subthreshold bipolar features. In addition to this, almost all clinical predictors of good response to lithium/atypical antipsychotics/antiepileptics are classical bipolar markers (familial bipolarity, eraly onset, intradepressive hypomanic symptoms, agitation, cyclothymic tempermanent, shorter episodes, more than three depressive episodes, and suicidality).

**Conclusion:**

Considering that lithium, atyipcal antipsychotics and antiepileptics, but not thyroid stimulating drugs have more and less antimanic effect, these results suggst that tretating intradecpressive hypomanic symptoms in bipolar mixed depression is a new (if not the only) explanation among the several previously proposed mechanisms of action of successful psychotropic augmentation of antidepressants in patients with MDE.

**Disclosure of Interest:**

None Declared

